# Supplementary material for: Balanced and positively worded personality short-forms: Mini-IPIP validity and cross-cultural invariance
Source: PeerJ. 2018 Sep 13;6:e5542. doi: 10.7717/peerj.5542 (PMC6139243; doi:10.7717/peerj.5542)
Supplement: Appendix A — Note. Mini-IPIP Spanish was adapted from Donellan et al., (2006); E, Extraversion; A, Agreeableness; C, Conscientiousness; N, Neuroticism; O, Openness; #, Item administration order; R, Reverse scored (recoded); nChilean = 518; n−ChileanPW = 278; nU.S. = 367. [file peerj-06-5542-s002.docx]

**Appendix A.**

The Mini-IPIP Scales Spanish and English versions (Chilean and U.S. descriptives)

| # | Factor | Facet | R | Wording (**Spanish**, *Spanish Positive*, Original) | M | SD | SK- | K- |
| --- | --- | --- | --- | --- | --- | --- | --- | --- |
| 1 | E | Cheerfulness |  | **Soy el alma de la fiesta** | 2.64 | 1.05 | 0.08 | –0.49 |
|  |  |  |  | Am the life of the party | 2.25 | 1.01 | 0.59 | –0.28 |
| 6 |  | Friendliness | R | **No hablo mucho** | 3.47 | 1.19 | –0.39 | –0.89 |
|  |  |  |  | *Hablo mucho* | 3.36 | 1.13 | –0.21 | –0.96 |
|  |  |  | R | Don’t talk a lot | 2.76 | 1.26 | 0.23 | –1.00 |
| 11 |  | Friendliness |  | **En las fiestas hablo con muchas personas** | 3.14 | 1.14 | –0.26 | –0.78 |
|  |  |  |  | Talk to a lot of different people at parties | 2.99 | 1.05 | 0.19 | –0.54 |
| 16 |  | Friendliness | R | **Prefiero pasar desapercibido** | 2.59 | 1.17 | 0.31 | –0.94 |
|  |  |  |  | *Me gusta ser el centro de atención* | 2.45 | 0.97 | 0.54 | 0.06 |
|  |  |  | R | Keep in the background | 2.58 | 1.13 | 0.47 | –0.65 |
| 2 | A | Sympathy |  | **Soy sensible hacia las emociones de otros** | 3.92 | 0.92 | –1.21 | 1.72 |
|  |  |  |  | Sympathize with others’ feelings | 4.09 | 0.77 | –0.99 | 1.69 |
| 7 |  | Sympathy | R | **No me interesan los problemas de otras personas** | 4.12 | 0.92 | –1.16 | 1.39 |
|  |  |  |  | *Me intereso por los problemas de otras personas* | 3.96 | 0.78 | –1.21 | 2.65 |
|  |  |  | R | Am not interested in other people’s problems | 3.69 | 0.92 | –0.64 | –0.04 |
| 12 |  | Sympathy |  | **Siento las emociones de los otros** | 3.70 | 0.91 | –0.71 | 0.48 |
|  |  |  |  | Feel others’ emotions | 3.69 | 0.90 | –0.74 | 0.57 |
| 17 |  | Sympathy | R | **En realidad no estoy interesado en los demás** | 4.19 | 0.86 | –1.13 | 1.41 |
|  |  |  |  | *En realidad me intereso por los demás* | 3.98 | 0.76 | –1.15 | 2.55 |
|  |  |  | R | Am not really interested in others | 3.82 | 0.93 | –0.65 | –0.09 |
| 3 | C | Dutifulness |  | **Realizo mis tareas inmediatamente** | 2.71 | 0.99 | 0.33 | –0.58 |
|  |  |  |  | Get chores done right away | 2.84 | 1.07 | 0.24 | –0.89 |
| 8 |  | Orderliness | R | **A menudo olvido poner las cosas en su lugar** | 3.15 | 1.25 | –0.07 | –1.19 |
|  |  |  |  | *Suelo poner las cosas en su lugar* | 3.27 | 1.06 | –0.29 | –1.01 |
|  |  |  | R | Often forget to put things back in their proper place | 3.31 | 1.17 | –0.16 | –1.13 |
| 13 |  | Orderliness |  | **Me gusta el orden** | 3.68 | 1.02 | –0.69 | –0.05 |
|  |  |  |  | Like order | 3.82 | 0.86 | –0.57 | 0.07 |
| 18 |  | Orderliness | R | **Soy desordenado** | 3.18 | 1.29 | –0.09 | –1.14 |
|  |  |  |  | *Soy ordenado* | 3.19 | 1.15 | –0.06 | –1.04 |
|  |  |  | R | Make a mess of things | 3.52 | 1.10 | –0.42 | –0.65 |
| 4 | N | Anger |  | **Tengo frecuentes cambios de ánimo** | 2.64 | 1.15 | 0.35 | –0.78 |
|  |  |  |  | *Tengo pocos cambios de ánimo* | 2.76 | 1.01 | 0.49 | –0.66 |
|  |  |  |  | Have frequent mood swings | 2.71 | 1.19 | 0.24 | –1.00 |
| 9 |  | Anxiety | R | **Estoy relajado la mayor parte del tiempo** | 2.71 | 1.11 | 0.36 | –0.65 |
|  |  |  | R | Am relaxed most of the time | 2.61 | 1.02 | 0.38 | –0.58 |
| 14 |  | Anger |  | **Me molesto fácilmente** | 2.76 | 1.17 | 0.15 | –1.00 |
|  |  |  |  | *Difícilmente me molesto* | 2.92 | 1.13 | 0.04 | –1.04 |
|  |  |  |  | Get upset easily | 2.92 | 1.21 | 0.13 | –1.03 |
| 19 |  | Depression | R | **Rara vez me siento triste** | 2.72 | 1.11 | 0.42 | –0.75 |
|  |  |  | R | Seldom feel blue | 3.30 | 1.17 | –0.30 | –0.83 |
| 5 | O | Imagination |  | **Tengo mucha imaginación** | 4.04 | 0.98 | –0.92 | 0.26 |
|  |  |  |  | Have a vivid imagination | 3.77 | 1.04 | –0.68 | –0.17 |
| 10 |  | Intellect | R | **No estoy interesado en las ideas abstractas** | 3.73 | 1.02 | –0.47 | –0.52 |
|  |  |  |  | *Me interesan las ideas abstractas* | 3.61 | 1.05 | –0.38 | –0.57 |
|  |  |  | R | Am not interested in abstract ideas | 3.71 | 0.99 | –0.54 | –0.44 |
| 15 |  | Intellect | R | **Tengo dificultad para entender ideas abstractas** | 3.63 | 0.94 | –0.40 | –0.43 |
|  |  |  |  | *Entiendo con facilidad las ideas abstractas* | 3.58 | 0.91 | –0.51 | –0.12 |
|  |  |  | R | Have difficulty understanding abstract ideas | 3.71 | 1.00 | –0.55 | –0.40 |
| 20 |  | Imagination | R | **No tengo buena imaginación** | 4.02 | 1.03 | –1.01 | 0.36 |
|  |  |  |  | *Tengo buena imaginación* | 4.14 | 0.84 | –1.06 | 1.27 |
|  |  |  | R | Do not have a good imagination | 3.92 | 1.02 | –0.84 | 0.12 |

*Note.* Mini-IPIP Spanish was adapted from Donellan et al., (2006); E = Extraversion; A = Agreeableness; C = Conscientiousness; N = Neuroticism; O = Openness; # = Item administration order; R = Reverse scored (recoded); n_Chilean_ = 518, n_ChileanPW_ = 278, n_U.S._ = 367.
