# Supplementary material for: Balanced and positively worded personality short-forms: Mini-IPIP validity and cross-cultural invariance
Source: PeerJ. 2018 Sep 13;6:e5542. doi: 10.7717/peerj.5542 (PMC6139243; doi:10.7717/peerj.5542)
Supplement: Appendix B [file peerj-06-5542-s003.docx]

**Appendix B**

**Mini-IPIP scales Spanish versions**

**Instrucciones**

A continuación, se presenta una serie de frases cortas que describen cómo las personas se comportan generalmente. Léelas con atención y señala la alternativa de respuesta que mejor describa tu comportamiento en general. No hay respuestas correctas o incorrectas, y recuerda que los datos serán tratados con confidencialidad.

**Opciones de respuesta**

1 = Nada de acuerdo

2 = Algo de acuerdo

3 = Medio acuerdo

4 = Mucho acuerdo

5 = Total acuerdo

| # | Mini IPIP Spanish version |  | # | Mini IPIP Spanish Positive version |
| --- | --- | --- | --- | --- |
| 1 | Soy el alma de la fiesta |  | 1 | Soy el alma de la fiesta |
| 2 | Soy sensible hacia las emociones de otros |  | 2 | Soy sensible hacia las emociones de otros |
| 3 | Realizo mis tareas inmediatamente |  | 3 | Realizo mis tareas inmediatamente |
| 4 | Tengo frecuentes cambios de ánimo |  | 4 | Tengo pocos cambios de ánimo |
| 5 | Tengo mucha imaginación |  | 5 | Tengo mucha imaginación |
| 6 | No hablo mucho |  | 6 | Hablo mucho |
| 7 | No me interesan los problemas de otras personas |  | 7 | Me intereso por los problemas de otras personas |
| 8 | A menudo olvido poner las cosas en su lugar |  | 8 | Suelo poner las cosas en su lugar |
| 9 | Estoy relajado la mayor parte del tiempo |  | 9 | Estoy relajado la mayor parte del tiempo |
| 10 | No estoy interesado en las ideas abstractas |  | 10 | Me interesan las ideas abstractas |
| 11 | En las fiestas hablo con muchas personas |  | 11 | En las fiestas hablo con muchas personas |
| 12 | Siento las emociones de los otros |  | 12 | Siento las emociones de los otros |
| 13 | Me gusta el orden |  | 13 | Me gusta el orden |
| 14 | Me molesto fácilmente |  | 14 | Difícilmente me molesto |
| 15 | Tengo dificultad para entender ideas abstractas |  | 15 | Entiendo con facilidad las ideas abstractas |
| 16 | Prefiero pasar desapercibido |  | 16 | Me gusta ser el centro de atención |
| 17 | En realidad no estoy interesado en los demás |  | 17 | En realidad me intereso por los demás |
| 18 | Soy desordenado |  | 18 | Soy ordenado |
| 19 | Rara vez me siento triste |  | 19 | Rara vez me siento triste |
| 20 | No tengo buena imaginación |  | 20 | Tengo buena imaginación |

*Note.* # = Item administration order.
