# Supplementary material for: Balanced and positively worded personality short-forms: Mini-IPIP validity and cross-cultural invariance
Source: PeerJ. 2018 Sep 13;6:e5542. doi: 10.7717/peerj.5542 (PMC6139243; doi:10.7717/peerj.5542)
Supplement: Appendix C — Note. PANAS C-10 was adapted from Damasio et al., (2013); PA, Positive Affect; NA, Negative Affect; #, Item administration order. [file peerj-06-5542-s004.docx]

**Appendix C**

**PANAS-C10 Spanish version**

**Instrucciones**

A continuación, se presenta una serie de palabras. Describen diferentes sentimientos y emociones. Lee con atención estas palabras y señala la alternativa de respuesta que mejor te describa en general, o la mayor parte del tiempo.

**Opciones de respuesta**

1 = Nada de acuerdo

2 = Algo de acuerdo

3 = Medio acuerdo

4 = Mucho acuerdo

5 = Total acuerdo

| # | Factor | item | M | SD | SK- | K- |
| --- | --- | --- | --- | --- | --- | --- |
| 1 | PA | Alegre | 3.77 | 0.64 | **–**0.44 | 0.51 |
| 2 |  | Animado | 3.65 | 0.67 | **–**0.29 | 0.04 |
| 3 |  | Contento | 3.77 | 0.68 | **–**0.49 | 0.46 |
| 4 |  | Entretenido | 3.41 | 0.83 | **–**0.36 | 0.20 |
| 5 |  | Feliz | 3.81 | 0.76 | **–**0.71 | 1.06 |
| 6 | NA | Humillado | 1.28 | 0.63 | 2.55 | 7.37 |
| 7 |  | Incómodo | 1.78 | 0.80 | 0.79 | 0.08 |
| 8 |  | Irritado | 1.97 | 0.97 | 0.80 | 0.02 |
| 9 |  | Dolido | 1.72 | 0.94 | 1.45 | 1.83 |
| 10 |  | Triste | 1.63 | 0.91 | 1.52 | 2.02 |

*Note.* PANAS C-10 was adapted from Damasio et al., (2013); PA = Positive Affect; NA = Negative Affect; # = Item administration order.
